# Soluble CD44 in oral rinses for the early detection of cancer: a prospective cohort study in high-risk individuals

**DOI:** 10.1186/s12903-024-04463-8

**Published:** 2024-07-19

**Authors:** Shahm Raslan, Drew H. Smith, Isildinha M. Reis, Sophia J. Peifer, Garrett Forman, Uche C. Ezeh, Priyashma Joshi, Margaret Koester, Isabella Buitron, Abdurrahman Al-Awady, Jerri Halgowich, Huaping Liu, Claudia Gordon, Monica Webb Hooper, Larissa Sweeny, Elizabeth J. Franzmann

**Affiliations:** 1 Department of Internal Medicine, Henry Ford Health, 2799 West Grand Boulevard, Detroit, MI 48202 USA; 2 Department of Otolaryngology - Head and Neck Surgery, Texas Tech University Health Sciences Center, 3601 4th St, Lubbock, TX 79430 USA; 3 Department of Public Health Sciences, University of Miami Leonard Miller School of Medicine, 1600 NW 10th Ave, Miami, FL 33136 USA; 4 Department of Otolaryngology – Head and Neck Surgery, University of Miami Leonard Miller School of Medicine, 1600 NW 10th Ave, Miami, FL 33136 USA; 5 New York University, New York, NY 10003 USA; 6 Sylvester Comprehensive Cancer Center, University of Miami Leonard Miller School of Medicine, 1475 NW 12th Ave, Miami, FL 33136 USA; 7 Case Comprehensive Cancer Center, Case Western Reserve University, 11100 Euclid Ave, Cleveland, OH 44106 USA; 8 Department of Otolaryngology – Head and Neck Surgery, Sylvester Comprehensive Cancer Center, 1475 NW 12th Ave, 310B, Miami, FL 33136 USA

**Keywords:** Early detection of Cancer, Cancer Biomarker, Oral rinse, Saliva, Oropharyngeal Cancer, Oral Cancer, Smoking

## Abstract

**Background:**

There are 54,000 new cases of oral cavity and oropharyngeal cancer in the United States and more than 476,000 worldwide each year. Oral cavity and oropharyngeal squamous cell carcinoma make up most tumors with five-year survival rates of 50% due to prevalence of late-stage diagnoses. Improved methods of early detection in high-risk individuals are urgently needed. We aimed to assess the tumorigenic biomarkers soluble CD44 (solCD44) and total protein (TP) measured using oral rinses as affordable convenient screening tools for cancer detection.

**Methods:**

In this prospective cohort study, we recruited 150 healthy current or former smokers through a community screening program. Baseline and four annual visits were conducted from March 2011-January 2016 with records followed until August 2020. Participants provided oral rinses, received head and neck exams, and completed questionnaires. SolCD44 and TP levels were measured and compared across groups and time. Participants were placed in the cancer group if malignancy developed in the study period, the suspicious group if physical exams were concerning for premalignant disease or cancer in the head and neck, and the healthy group if there were no suspicious findings. This analysis used two-sample t-test for comparison of means and two-sample Wilcoxon Test for comparison of medians. For subjects with follow-ups, estimated means of biomarkers were obtained from a fitted Repeated Measures Analysis of Variance (RANOVA) model including group, visit, and their interaction. Pairwise comparisons of mean solCD44 were made, including intergroup and intragroup comparison of values at different years.

**Results:**

Most participants were males (58.7%), < 60 years of age. (90.7%), and Black (100%). Baseline mean solCD44 was elevated (2.781 ng/ml) in the cancer group compared to the suspicious group (1.849 ng/ml) and healthy group (1.779 ng/ml).

**Conclusion:**

This study supports the feasibility of a CD44-based oral rinse test as an affordable and convenient adjunctive tool for early detection of aerodigestive tract and other cancers in high-risk populations.

**Supplementary Information:**

The online version contains supplementary material available at 10.1186/s12903-024-04463-8.

## Background

In 2022, there will be an estimated 54,000 new cases of OOPC in the US and more than 476,000 worldwide each year [1, 2]. Squamous cell carcinomas compromise over 90% of these cancers [3]. Five-year world-wide survival rates for oral cavity and oropharyngeal cancer are about 50% due to the prevalence of late-stage diagnoses [4, 5]. The incidence of late-stage head and neck cancer continues to increase especially in males and Black communities [6]. Low socioeconomic status (SES), homelessness, and low income are associated with increased incidence of OPSCC [7, 8]. Black race also is also associated with more advanced stage at presentation. Prevalence was associated with other SES-related factors as well including literacy and occupation [9, 10]. Early detection is critical to reduce mortality and combat disparities in these groups, as it increases survival rates to 80–90% [11].

Long-term studies of OOPSCC screening programs show reduced mortality in those diagnosed at early stages [12–14]. Screening programs targeted towards high-risk communities in particular have been effective. In Kerala, India, an oral exam screening program administered by trained health workers significantly reduced oral cancer mortality by 43% in men with tobacco or alcohol use, or both, in the intervention group compared with controls [12]. A population-based oral cancer screening program using oral exams by trained clinicians that targeted more than 2 million Taiwanese cigarette smokers and/or betel quid chewers demonstrated a 26% reduction in mortality in the screened group [13].

Due to early diagnostic potential and advantages in cost and availability, there is increased interest in developing salivary biomarker tests for head and neck cancer [15, 16]. CD44 protein is a cell-membrane-associated glycoprotein that facilitates interactions with the extracellular matrix, with active roles in immune responses [17, 18], inflammation, tumorigenesis, and metastases, especially after cleavage into its soluble form [19–22]. Increased CD44 levels are correlated with a wide range of cancers [20, 23]. Tobacco use, a principle high-risk exposure in the development of many cancers [20], has been correlated with levels of soluble CD44 (solCD44) in saliva in a smoking cessation study by our group [24, 25]. Our group previously demonstrated both solCD44 and total protein (TP) levels in combination are elevated in salivary samples from head and neck squamous cell carcinoma (HNSCC) patients compared to controls, and were able to distinguish HNSCC from controls with 62–79% sensitivity and 88–100% specificity. A point of care test utilizing solCD44 and TP levels distinguished OOPSCC cases from controls with 90% sensitivity and 62% specificity. This suggests these biomarkers may be useful for clinical decision-making.

In the previously discussed smoking cessation study, our group assessed a low-income and high-risk cohort of 150 subjects recruited through a screening program in South Florida. One aim of the study included a 1-year smoking cessation program which was associated with significant drops in solCD44 levels [25], while the solCD44 and TP lab test had a specificity of 74% at baseline evaluation [11]. For our current study, we follow these subjects over time. The objective of this study is to evaluate solCD44 and TP levels in 150 participants with high cancer risk during annual visits over 4 years and to follow prospectively to determine if CD44 and TP are effective early biomarkers for OOPSCC and other cancers.

## Patients and methods

### Study design and enrollment

This prospective cohort study enrolled 150 participants from 2011 to 2016. Enrollment was initiated through a community-based outreach partnership in South Florida. Subjects were recruited with convenience sampling at a high-volume food bank and a public housing project. This study aimed to work with known high-risk populations who suffer disproportionately from these malignancies. Subjects were included if they were high-risk for development of malignancy with ≥ 100 lifetime cigarettes and ≥ 21 years of age, although subjects > 40 years were given preference considering rarity of malignancy in younger groups. 139/150 (92.6%) of subjects were active smokers at the baseline visit. Subjects with prior premalignancy or cancer were excluded. A sample size of 150 was selected to obtain 30 participants with full smoking cessation within 3 months for a separate aim of the study. Smoking is a known cause of disease, so the cessation program was not withheld from participants for ethical reasons despite potential confounding.

### Study visits and data collection

Participants meeting inclusion criteria completed study visits at baseline then annually for 4 years. For each visit, participants provided an oral rinse, received a head and neck physical exam with a board-certified head and neck surgeon, and completed a self-administered risk-assessment questionnaire. Oral rinses were collected using a previously published method that samples the oral cavity and oropharynx [26]. Patients placed 5 mL of normal saline in their mouths, swished for five seconds, gargled for five seconds, and then deposited the oral rinse into a specimen cup. Specimen were then transported on ice and stored at -80 degrees F. Finally, SolCD44 and TP levels were determined from the oral rinse specimens. Questionnaires documented demographics, oral health, risk-exposures (such alcohol and tobacco consumption), dietary habits, and other covariables. Oral health was described as either “poor/fair” or “good” based on categorization of self-reported variables (see Table 1). Subjects were stratified according to risk of malignancy based on physical exam findings. Further medical recommendations were made as appropriate. Subjects that warranted immediate specialist consultation for suspected neoplasm were noted and referred to participating clinics at the University of Miami and Jackson Memorial Hospital.

**Table 1:** Characteristics of participants at baseline

	*N*	%
**All**	150	100.0
**Age**		
<60	136	90.7
60 or more	14	9.3
**Gender**		
Male	88	58.7
Female	62	41.3
**Education**		
Grades 1–8	2	1.3
Grades 9–12	113	75.3
Some college	32	21.3
College graduate	3	2.0
**Employment**		
Occupation with some income	44	29.3
Out-of/unable-to work	106	70.7
**Income**		
≥$25,000	16	10.7
<$25,000	134	89.3
**SES**		
Low	150	100.0
**Oral health** ^**a**^		
Poor/Fair	68	45.3
Good	82	54.7
**Teeth removed** ^**b**^		
NA	2	1.3
None/1–5	69	46.0
6+, but not all	67	44.7
All	12	8.0
**Drinking habits**		
Non-drinker/Mild	30	20.0
Moderate/Heavy	120	80.0
**Drinking habits (SR Def.)**		
Non-drinker/Mild	40	26.7
Moderate/Heavy	110	73.3

^a^Oral Health Score: Poor/Fair: if gingivitis or periodontitis or other oral health pathology currently present OR if last visit to dentist/dental clinic 5 or more years ago OR if last cleaning by dental hygienist was 5 or more years ago OR if teeth brushed less than once a day. Good: if no gingivitis or periodontitis or other oral health pathology currently present AND if last visit to dentist/dental clinic less than 5 years ago AND if last cleaning by dental hygienist was less than 5 years ago AND if teeth brushed more than once a day

^b^Teeth removed included those removed due to infection or decay, and excluded teeth removed due to injury or for orthodontic intervention

### Assays

Participants provided oral rinse samples at every visit. solCD44 was quantified with a sandwich-ELISA assay (eBioscience, San Diego, CA, USA). TP was quantified with the DC protein assay (Bio Rad Laboratories, Hercules, CA, USA). Assays were prepared according to protocols as previously published [12, 16, 20, 26–29]. Lab technicians were blinded to subjects’ data and completed assays in the lab of Dr. Elizabeth Franzmann at the University of Miami Department of Otolaryngology. In our prior work, SolCD44 was considered high-risk for malignancy if > = 2.22 and < 5.33 ng/mL depending on TP, or if > 5.33 ng/mL regardless of TP [11]. However, these cut-points were not applied in the current work.

### Follow-Up

Participants and their medical records were followed for 9 years from study enrollment until August 30th, 2020 using IRB-approved protocols. Records were reviewed from Jackson Memorial Hospital, a county hospital, and the University of Miami Hospital and Clinics, a private institution, both serving the study population. Subjects who developed malignancy during follow-up were placed into the cancer group. Subjects with head and neck lesions suspicious for premalignancy or malignancy on physical exam were referred for immediate follow-up and placed in the suspicious group. Subjects with diagnosed malignancy in the study window were placed in the cancer group. All remaining subjects who did not have suspicious lesion or malignancy were placed in the “healthy” group, though they were still at risk due to habits. Subjects were given a gift card after completing each visit. Days between visit and cancer diagnoses were recorded.

### Statistical analysis

SolCD44 and TP levels were compared between groups and over time. Subjects without follow-up visits were excluded. To assess for potential bias introduced by excluding subjects without follow-up visits, biomarker levels of these participants were compared with levels of those with follow-up visits. This analysis used two-sample t-test for comparison of means and two-sample Wilcoxon Test for comparison of medians. For subjects with follow-ups, estimated means of biomarkers were obtained from a fitted Repeated Measures Analysis of Variance (RANOVA) model including group, visit, and their interaction. A second RANOVA model including number of current cigarettes and age (categorized as < 50 or ≥ 50) was fitted to assess for confounding. Pairwise comparisons of mean solCD44 were made with unadjusted and False Discovery Rate (FDR) adjusted *p*-values using both described models. Selected comparisons included intergroup comparison of values at baseline, year 1, and year 4 between all groups (healthy vs. suspicious, healthy vs. cancer, and suspicious vs. cancer). Intragroup comparisons (i.e., healthy vs. healthy) were done for baseline vs. year 1, year 1 vs. year 2, and baseline vs. year 4. All comparisons were considered significant at *p* ≤ 0.05. Statistical analyses were performed using SAS version 9.4 (SAS Institute, Inc., Cary, NC, USA).

## Results

One hundred and fifty subjects were enrolled in the study and completed baseline visits, with characteristics reported in Table 1.

One hundred twenty-six subjects remained healthy according to electronic medical record review at end of the study period (August 2020). Nine subjects developed malignancy in this period and were retrospectively categorized as the cancer group. Seven of the 9 patients followed up at the year 1 visit, 6 of the 9 followed up in year 2, 5 of the 9 followed up in year 3, and 4 of the 9 followed up in year 4. These cancer cases included 4 squamous cell carcinomas (one tongue, one lung, and two esophagus), and 5 additional cancers (one each lung adenocarcinoma, pancreatic adenocarcinoma, hepatocellular carcinoma, malignant phyllodes breast tumor, and endometrial verses cervical mesonephric adenocarcinoma).

Fifteen subjects had physical exam findings highly suspicious of neoplasm during study visits and were categorized in the suspicious group. These findings are summarized in Additional Table 1. One additional suspicious case developed tongue cancer and was placed in the cancer category. Ninety-four subjects (63%) followed up at year 1 (76 healthy, 11 suspicious, 7 cancer). Seventy-five (50%) followed up at year 2 (64 healthy, 5 suspicious, 6 cancer). Fifty-six (37%) followed up at year 3 (46 healthy, 5 suspicious, 5 cancer). Twenty-seven (18%) followed up at year 4 (20 healthy, 3 suspicious, 4 cancer).

**Fig. 1:**
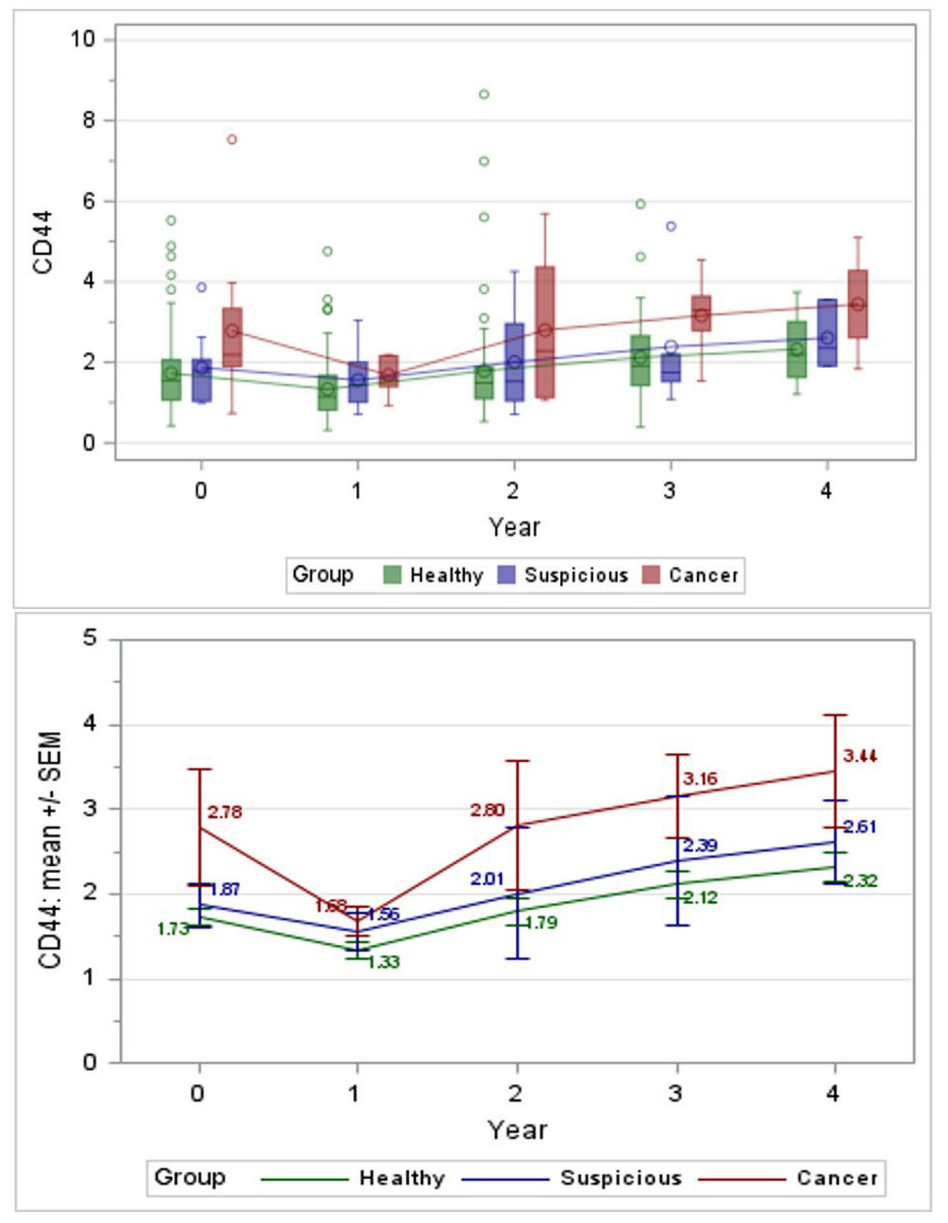
Boxplots and Mean solCD44 (ng/mL) in Subjects with Baseline and ≥ 1 Follow-Up Visit

One hundred four subjects had at least 1 follow-up visit and thus were included in further statistical analysis. Figure 1 shows mean solCD44 in subjects with baseline and ≥ 1 follow-up visit. Pairwise comparisons of mean solCD44 levels were made for specific group comparisons at specific visits (baseline, years 1 and 4) or between visits (baseline, year 1, 2, and 4) or within a group. Five of 18 comparisons showed significant differences (Table 2).

**Table 2:** Unadjusted and FDR-adjusted *p*-values For Selected Comparisons of Estimated solCD44 Means

Group 1		Group 2		Mean		Raw-*p*	FDR-*p*
Visit	Disease Status	Visit	Disease Status	Difference^a^	SE		
Baseline	Cancer	Baseline	Healthy	+ 1.0530	0.3828	0.0070	**0.0252**
Baseline	Cancer	Baseline	Suspicious	+ 0.9144	0.4905	0.0651	0.1464
Baseline	Suspicious	Baseline	Healthy	+ 0.1385	0.3499	0.6930	0.7752
Year 1	Cancer	Year 1	Healthy	+ 0.3456	0.2947	0.2435	0.3831
Year 1	Cancer	Year 1	Suspicious	+ 0.1257	0.3663	0.7322	0.7752
Year 1	Suspicious	Year 1	Healthy	+ 0.2199	0.2504	0.3820	0.4583
Year 4	Cancer	Year 4	Healthy	+ 1.0157	0.4982	0.0521	0.1339
Year 4	Cancer	Year 4	Suspicious	+ 0.9070	0.6905	0.2008	0.3831
Year 4	Suspicious	Year 4	Healthy	+ 0.1087	0.5525	0.8456	0.8456
Year 1	Cancer	Baseline	Cancer	-1.0945	0.3415	0.0017	**0.0101**
Year 2	Cancer	Year 1	Cancer	+ 1.0839	0.5111	0.0364	0.1093
Year 4	Cancer	Baseline	Cancer	+ 0.6371	0.5732	0.2707	0.3831
Year 1	Suspicious	Baseline	Suspicious	-0.3058	0.2926	0.2980	0.3831
Year 2	Suspicious	Year 1	Suspicious	+ 0.6533	0.5858	0.2679	0.3831
Year 4	Suspicious	Baseline	Suspicious	+ 0.6445	0.6034	0.2908	0.3831
Year 1	Healthy	Baseline	Healthy	-0.3871	0.1081	0.0005	**0.0073**
Year 2	Healthy	Year 1	Healthy	+ 0.5251	0.1517	0.0008	**0.0073**
Year 4	Healthy	Baseline	Healthy	+ 0.6744	0.2260	0.0045	**0.0204**

^a^Group 1 minus Group 2 mean difference comparing two disease status groups at a fixed visit or two visits within a particular group of subjects; estimated mean differences and standard errors (SEs) from RANOVA model including group, visit, and group×visit interaction. Bold: the CD44 mean difference is statistically significant different from zero (FDR-*p* < 0.05)

Subjects that developed cancer had significantly higher baseline solCD44 levels compared to healthy subjects (cancer minus healthy mean difference + 1.053 ng solCD44, FDR-*p* = 0.0252), raising the possibility that elevated solCD44 levels may predict future development of cancer. Other significant findings included: year 1 cancer group had lower levels than baseline cancer group (year 1 minus baseline mean − 1.0945, FDR-*p* = 0.0101), year 1 healthy subjects had lower levels than baseline healthy subjects (difference − 0.387, FDR-*p* = 0.0073), year 2 healthy subjects had higher levels than year 1 healthy subjects (+ 0.525, *p* = 0.0073), and year 4 healthy subjects had higher levels than baseline healthy subjects (+ 0.6744, *p* = 0.0204). Since subjects were provided smoking-cessation resources and aged during the study, we included tobacco and age in a post-hoc multivariable model using categories < 50 or ≥50 and cigarettes per day at present. Results revealed no confounding (Additional Table 2). Periodontal disease and oral health were also assessed for potential confounding, though results showed no significant associations (Additional Tables 3 and 4). Furthermore, there was no significant difference in baseline solCD44 values between subjects with (*n* = 104) and without (*n* = 46) follow-up; mean = 1.83 ng/mL (SD = 1.11) vs. 1.87 ng/mL (SD = 0.85), respectively, *p* = 0.814.

Many subjects did not follow-up every year so cohorts of years 1, 2, 3, and 4 did not always include the same subjects. Therefore, we also compared mean solCD44 levels for subjects who followed up each year paired with their own levels at baseline (Additional Table 5). There were significant differences from baseline in both overall (all subjects) and healthy groups for year 1 (overall − 0.423 ng/ml, *p* < 0.001; healthy group − 0.371 ng/ml, *p* < 0.001); year 3 (overall, + 0.416 ng/ml, *p* = 0.019; healthy group + 0.436 ng/ml, *p* = 0.010); and year 4 (overall + 0.719 ng/ml, *p* = 0.003; healthy group + 0.613 ng/ml, *p* = 0.012). Notably, with only 4 subjects, levels in the cancer group nearly doubled over the course of the study, though these results fell just short of significance (*p* = 0.083).

Table 3 shows the time in months from baseline and from final visit dates to cancer diagnoses.

**Table 3:** Cancer group, time to diagnosis from visit dates, and tnm stage at diagnosis

Diagnosis	Months from Baseline to Diagnosis	Date of Diagnosis Minus Date of Last Visit (Months)	CD44 Baseline	CD44 Year 1	CD44 Year 2	CD44 Year 3	CD44 Year 4	TNM Stage
Hepatocellular Carcinoma	8.1	(17.6) ^*a*^	1.08	− ^*b*^	1.940	−	−	T2NXM0
Malignant Phyllodes Breast Tumor	12.5	(2.9)	3.34	1.575	−	−	−	T3N0M0
Lung Squamous Cell Carcinoma	15.0	(8.6)	3.975	−	4.365	−	−	T1N3M0
Lung Adenocarcinoma	33.0	(12.2)	0.73	0.928	1.060	1.540	3.38	T3N2M0
Pancreatic Adenocarcinoma	54.6	19.4	7.535	2.190	−	2.790	−	T4NXM0
Esophageal Squamous Cell Carcinoma	60.8	15.0	2.265	2.030	5.690	4.545	5.1	T4N0M0
Endometrial Carcinoma	77.4	62.9	2.19	1.4	−	−	−	T4NXM1
Esophageal Carcinoma	79.5	31.2	2.02	2.162	1.13	3.64	3.45	T1N0M0
Tongue Squamous Cell Carcinoma	83.8	37.8	1.895	1.495	2.625	3.295	1.835	T2N0M0

^a^(): denotes negative number for diagnosis before last visit. ^b^Blank cells (-): no CD44 value; participant missed specific visit

**Fig. 2:**
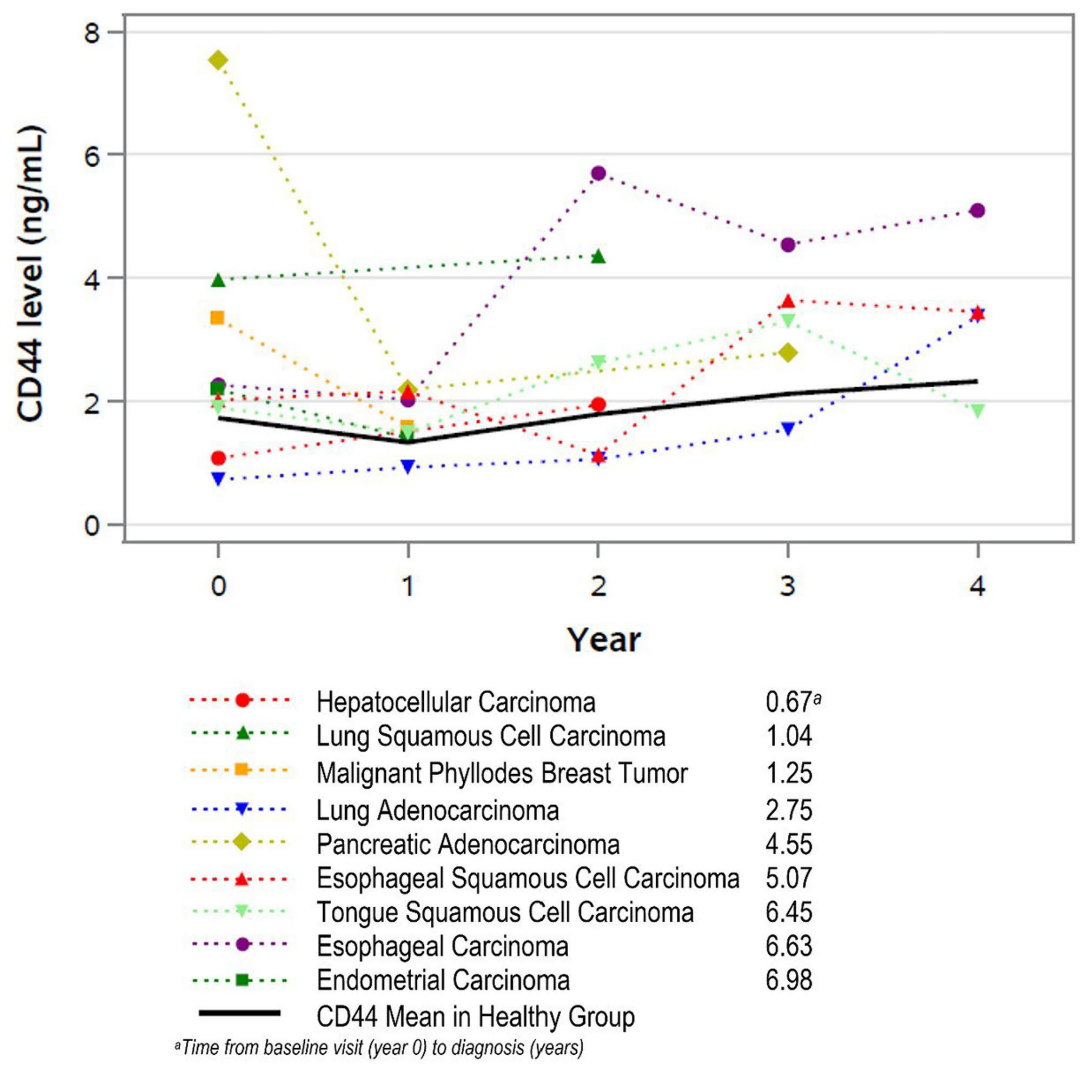
Cancer group, individual solcd44 levels over time, time of diagnosis, and mean of the healthy group

Nine cancer cases were detected with mean 47.2 months from baseline visit to cancer diagnosis. The earliest cancer diagnosis (hepatocellular carcinoma) was made 8.1 months after baseline (17.6 months before the subject’s last study visit). Three additional cases were diagnosed during the study at 12.5, 15, and 33 months from baseline. In these 4 cases, diagnosis was at mean 10.3 months before last study visit, and they had an average solCD44 of 2.484 ng/ml on the visit immediately preceding diagnosis. Five cases were diagnosed after conclusion of the study with mean 33.3 months between the last study visit and cancer diagnosis. Four were diagnosed more than 6 years from baseline, at 15, 31.2, 37.8 and 62.9 months after the last visit. These four cases had a mean solCD44 of 2.946 ng/ml at the final visit (two were solCD44 values 3.45 and 5.1). Eight of the 9 subjects with cancer had study visits within 3 years of diagnosis. These results are shown graphically for individual cancer cases with the healthy mean solCD44 superimposed in a solid black line (Fig. 2).

For all subjects with at least 1 follow-up visit, we assessed TP levels for all groups. Additional Fig. 1 shows mean TP in subjects with baseline and ≥ 1 follow-up visit. Pairwise comparisons were made of mean TP levels for specific groups at specific visits. No comparisons showed significant differences when analyzing with and without adjustment for age and tobacco use (Additional Tables 6 and 7). Interestingly, TP levels in the suspicious and cancer groups followed more closely together (Additional Fig. 1), whereas solCD44 levels in healthy and suspicious groups followed more closely together (Fig. 1). Differences in TP between healthy and suspicious groups were most pronounced at years 1 and 4 and between healthy and cancer groups at year 4, though none reached statistical significance.

## Discussion

Convenient, noninvasive tools for the earlier detection of OOPSCC and other malignancies are urgently needed, especially in high-risk groups facing disparities of care. This study assessed whether oral rinses quantifying solCD44 and TP levels are useful early detection tools for cancer. We found elevated solCD44 preceded clinical findings in those that developed malignancies.

Early detection is critical for earlier interventions which reduce morbidity and mortality in cancer treatment. The premalignant stage offers a window of opportunity where lifestyle modifications reduce the need for cancer treatments including surgery altogether. Our group previously found decreased solCD44 levels associated with increased green salad intake and found levels decreased following tobacco cessation [30]. Future work should focus on CD44 as a monitoring tool before and after behavioral modification.

In this pilot study, the cancer group had significantly higher mean solCD44 levels than the healthy group at baseline. The cancer group was diagnosed with malignancy 3.93 years on average after their baseline visit. This is the first study showing elevated solCD44 and clinical findings in parallel and showing solCD44 levels leading up to cancer diagnoses. Our study confirms previous studies showing solCD44 in oral rinses as a sensitive and cost-effective marker of early tumorigenesis [11, 16, 25, 27–29]. Three of the 4 cancers diagnosed during the study were tobacco-related malignancies with solCD44 levels rising at time of diagnosis. The malignant phyllodes tumor of the breast diagnosed in the study showed decreasing levels possibly because it is not a tobacco-related malignancy. Since all the cancers detected, except for the endometrial cancer and malignant phyllodes tumor, are associated with tobacco use, the role of CD44 as an early detection tool for tobacco users can extend beyond OOPSCC. Further study should investigate whether clinicians should increase surveillance among smokers with rising solCD44 levels. Low-dose CT scans in smokers have been shown to significantly reduce the risk of death due to lung cancer [31]. With further study, this and other recommended screening tests such as colonoscopy, pap smears and mammography may be triggered by rising solCD44. In the future, additional testing that is not currently routinely recommended may also be warranted such as laryngoscopy, esophagoscopy, and liver function tests or ultrasound.

In the cancer group, solCD44 levels rose significantly over time and 7 of the 9 cancers that developed were tobacco-related. While increased solCD44 levels among those who developed cancer were not confounded by tobacco use, it is possible that these increases were affected by increased alcohol use, weight gain, or dietary changes, as these are other fluctuating risk-factors.

solCD44 levels likely correlate with increased plasma levels, as saliva contains transudate from the blood [28]. As non-head and neck tumors are highly vascular and upregulate numerous serum proteins, it is likely that these tumors leak proteins such as IgG and albumin from the serum into the oral rinse samples, thus raising solCD44 levels.

A significant drop in solCD44 occurred at the year 1 visit in the healthy and cancer groups compared to baseline. In this period, a subset of our cohort was simultaneously enrolled in a one-year smoking cessation program as part of another arm of our study. Smoking cessation was considered a potential confounder accounting for the drop in solCD44, as suggested by previous studies. Similarly, average solCD44 increased over time for all groups, raising the consideration of confounding by increased age. However, in a second multivariate model accounting for cigarette consumption and increased age, there were negligible statistical effects, as shown in Additional Tables 2 and 7. Therefore, drops in year 1 are potentially explained by education and increased awareness about cancer and risk-exposures in participants after enrollment. This highlights an added benefit of closer follow-ups in higher-risk groups in addition to those of regularly monitoring solCD44 levels. Moreover, no comparisons performed among the cohort groups showed significant TP level differences, though there was suggestion of higher TP levels for both the suspicious and cancer group at year 4. Further study on CD44 and TP levels in larger high-risk cohorts is needed to confirm these findings.

Sixty-three percent of the cohort followed up in year 1, followed by 50% in year 2, 37% in year 3, and 18% in year 4. These are higher follow-up rates year-to-year than similar under-resourced community screening studies, which ranged from 28 to 38% for various study designs and follow-up conditions [32–36]. The low SES and older age of our cohort likely increased attrition while the gift cards given at follow-ups contributed to participant retention [38, 39]. Furthermore, 9 of 150 participants, or 6% of our high-risk cohort, developed cancer during our 10-year study window. This rate is in line with projected expectations of 5.4%, based on the 2022 national annual rate of new cancer diagnosis in non-Hispanic Blacks of 0.54% [40].

This pilot study has limitations. Subjects received only head and neck physical exams which omitted findings in other systems. While efforts were made to create an exhaustive questionnaire, valuable historical data may still have been missed. Recall bias is also a known limitation of self-report questionnaires. In addition, enrollment in our study relied on convenience sampling which is prone to volunteer bias. Our sample was also selectively high-risk and without heterogeneity. Furthermore, cancer development was determined via electronic medical records review at two main hospitals serving this population after completion of the study. A minority of subjects had records at outside institutions that were unfortunately unavailable. Follow up at the fourth visit was lower than expected at 18% compared to baseline. Robust measures to minimize attrition rates over multiple years and to ensure availability of medical records should be considered to obtain valuable follow-up data. Efforts to retain communication with subjects that travel or move away should also be made.

Future investigations with larger cohorts should be performed. Clinicians from multiple specialties should participate in future studies to correlate solCD44 levels with exam findings outside the head and neck region, considering the variety of malignancies developed in our cohort. Prospective studies should be performed focusing on smoking-related cancers other than just OOPSCC. In-depth reporting on the cancer group of this study with timelines of exam findings, histories, and biomarker levels would yield valuable insight as well.

## Conclusion

SolCD44 levels were increased leading up to cancer diagnosis. This was the first study to compare risk-exposures, clinical findings, and solCD44 levels in individual subjects before cancer development. In high-risk groups facing disparities of care, close monitoring of solCD44 levels may provide valuable insight into subclinical premalignant changes at the individual level. Oral rinses quantifying solCD44 are cost-efficient and noninvasive adjuncts to regular screenings. Oral rinse solCD44 levels may identify patients at high risk for developing smoking-related cancers. Larger prospective studies involving more centers are warranted.

### Electronic supplementary material

Below is the link to the electronic supplementary material.

Supplementary Material 1

## Data Availability

The datasets generated and/or analyzed during the current study are not publicly available to maintain the privacy of participants. Data are available from the corresponding author on reasonable request and with the permission of the University of Miami.

## Abbreviations

FDR: False Discovery Rate
HNSCC: Head and neck squamous cell carcinoma
OOPSCC: Oral cavity and oropharyngeal squamous cell carcinoma
RANOVA: Repeated Measures Analysis of Variance
SES: Socioeconomic status
solCD44: Soluble CD44
TP: Total protein
