# Supplementary material for: Soluble CD44 in oral rinses for the early detection of cancer: a prospective cohort study in high-risk individuals
Source: BMC Oral Health. 2024 Jul 19;24:820. doi: 10.1186/s12903-024-04463-8 (PMC11264816; doi:10.1186/s12903-024-04463-8)
Supplement: Supplementary file 1 — Supplementary Material 1 [file 12903_2024_4463_MOESM1_ESM.docx]

**Additional Files**

| **Additional Table 1. Clinical Findings of 24 Physical Exams With Suspicious Findings Requiring Urgent Follow-Up** | | |
| --- | --- | --- |
|  | **N** | **%** |
| *Total* |  |  |
| **Finding** | 24 | 100% |
| *Oral leukoplakia* | 7 | 46.67 |
| *Lymphadenopathy* | 5 | 33.3 |
| *Laryngeal polyp* | 3 | 20 |
| *Oral leukoplakia with erythema* | 2 | 13.3 |
| *Base of tongue mass* | 2 | 13.3 |
| *Cranial nerve neuropathy* | 1 | 6.67 |
| *Erythroplasia* | 1 | 6.67 |
| *Hoarseness* | 1 | 6.67 |
| *Papillomatous lip lesion* | 1 | 6.67 |
| *Frictional keratosis* | 1 | 6.67 |
| **Participant Disposition:** |  |  |
| *Total* | 15 | 100% |
| *Lost to follow up* | 11 | 73.3 |
| *Average number follow up visits* | 1.67 |  |
|  | | |

| **Additional Table 2. Unadjusted and FDR-adjusted p-values For Selected Comparisons of Estimated solCD44 Means.** | | | | | | | |
| --- | --- | --- | --- | --- | --- | --- | --- |
| *Group 1* | | *Group 2* | | *Mean* | |  |  |
| *Visit* | *Disease Status* | *Visit* | *Disease Status* | *Difference^a^* | *SE* | *Raw-p* | *FDR-p* |
| *Baseline* | *Cancer* | *Baseline* | *Healthy* | +1.0457 | 0.3860 | 0.0079 | **0.0293** |
| *Baseline* | *Cancer* | *Baseline* | *Suspicious* | +0.9156 | 0.4925 | 0.0658 | 0.1481 |
| *Baseline* | *Suspicious* | *Baseline* | *Healthy* | +0.1301 | 0.3542 | 0.7141 | 0.7873 |
| *Year 1* | *Cancer* | *Year 1* | *Healthy* | +0.3400 | 0.2992 | 0.2584 | 0.3870 |
| *Year 1* | *Cancer* | *Year 1* | *Suspicious* | +0.1213 | 0.3698 | 0.7436 | 0.7873 |
| *Year 1* | *Suspicious* | *Year 1* | *Healthy* | +0.2187 | 0.2516 | 0.3868 | 0.4642 |
| *Year 4* | *Cancer* | *Year 4* | *Healthy* | +1.0092 | 0.5020 | 0.0551 | 0.1417 |
| *Year 4* | *Cancer* | *Year 4* | *Suspicious* | +0.9037 | 0.6941 | 0.2047 | 0.3870 |
| *Year 4* | *Suspicious* | *Year 4* | *Healthy* | +0.1055 | 0.5560 | 0.8510 | 0.8510 |
| *Year 1* | *Cancer* | *Baseline* | *Cancer* | -1.1050 | 0.3474 | 0.0018 | **0.0151** |
| *Year 2* | *Cancer* | *Year 1* | *Cancer* | +1.0845 | 0.5117 | 0.0365 | 0.1096 |
| *Year 4* | *Cancer* | *Baseline* | *Cancer* | +0.6237 | 0.5801 | 0.2864 | 0.3870 |
| *Year 1* | *Suspicious* | *Baseline* | *Suspicious* | -0.3107 | 0.2956 | 0.2953 | 0.3870 |
| *Year 2* | *Suspicious* | *Year 1* | *Suspicious* | +0.6493 | 0.5874 | 0.2721 | 0.3870 |
| *Year 4* | *Suspicious* | *Baseline* | *Suspicious* | +0.6356 | 0.6079 | 0.3010 | 0.3870 |
| *Year 1* | *Healthy* | *Baseline* | *Healthy* | -0.3993 | 0.1301 | 0.0025 | **0.0151** |
| *Year 2* | *Healthy* | *Year 1* | *Healthy* | +0.5223 | 0.1525 | 0.0009 | **0.0151** |
| *Year 4* | *Healthy* | *Baseline* | *Healthy* | +0.6602 | 0.2406 | 0.0081 | **0.0293** |
| Analysis of means from RANOVA model accounting for tobacco and age as confounders  ^a^ Group 1 minus Group 2 mean difference comparing two disease status groups at a fixed visit or two visits within a particular group of subjects; estimated mean differences and standard errors (SEs) from RANOVA model including group, visit, group×visit interaction, and tobacco and age as confounders. Bold: the CD44 mean difference is statistically significant different from zero (FDR-p <0.05). | | | | | | | |

| **Additional Table 3. Associations between solCD44 Oral Health, and Teeth Removed at Baseline Visit** | | | | |
| --- | --- | --- | --- | --- |
|  |  | **CD44** |  |  |
|  | N | **Mean** | SD | SE |
| *All* | 150 | 1.85 | 1.04 | 0.08 |
| *Oral health* |  |  |  |  |
| *Poor/Fair* | 82 | 1.79 | 1.04 | 0.12 |
| *Good* | 68 | 1.91 | 1.03 | 0.13 |
| *P* |  | 0.476 |  |  |
| *Teeth removed^a^ (n=148)* |  |  |  |  |
| *None/1-5* | 69 | 1.90 | 0.91 | 0.11 |
| *6 or more/All* | 79 | 1.78 | 1.13 | 0.13 |
| *P* |  | 0.502 |  |  |
| *Teeth removed (n=148)* |  |  |  |  |
| *None/1-5* | 69 | 1.90 | 0.91 | 0.11 |
| *6+, but not all* | 67 | 1.88 | 1.18 | 0.14 |
| *All* | 12 | 1.23 | 0.59 | 0.17 |
| *P* |  | 0.103 |  |  |
| SD: standard deviation, SE: standard error. P: p value from two sample t-test or ANOVA.  ^a^Teeth removed included those removed due to infection or decay, and excluded teeth removed due to injury or for orthodontic intervention. | | | | |

| **Additional Table 4. Associations between solCD44 Oral Health, and Teeth Removed For Participants with Baseline Visit and At Least 1 Follow-up Visit** | | | | |
| --- | --- | --- | --- | --- |
|  |  | **CD44** |  |  |
|  | N | **Mean** | SD | SE |
| *All* | 104^a^ | 1.83 | 1.11 | 0.11 |
| *Oral health* |  |  |  |  |
| *Poor/Fair* | 55 | 1.73 | 1.13 | 0.15 |
| *Good* | 49 | 1.95 | 1.10 | 0.16 |
| *P* |  | 0.302 |  |  |
| *Teeth removed^b^ (n=103)^c^* |  |  |  |  |
| *None/1-5* | 49 | 1.87 | 0.92 | 0.13 |
| *6 or more/All* | 54 | 1.77 | 1.25 | 0.17 |
| *P* |  | 0.636 |  |  |
| *Teeth removed (n=103)^c^* |  |  |  |  |
| *None/1-5* | 49 | 1.87 | 0.92 | 0.13 |
| *6+, but not all* | 48 | 1.80 | 1.31 | 0.19 |
| *All* | 6 | 1.52 | 0.66 | 0.27 |
| *P* |  | 0.757 |  |  |
| SD: standard deviation, SE: standard error. P: p value from two sample t-test or ANOVA.  ^a^N=104, excluding 46 without follow-up visits ^b^Teeth removed included those removed due to infection or decay, and excluded teeth removed due to injury or for orthodontic intervention.  ^c^n=103, excluding 46 without follow-up visits and 1 additional due to incomplete survey response | | | | |

| Additional Table 5. Comparison of solCD44 Levels: Baseline and Follow-up Years Paired Data | | | | | |
| --- | --- | --- | --- | --- | --- |
| *Baseline or*  *Follow-up Visit* |  | **CD44  at baseline** | **CD44  at follow-up** | **Difference** |  |
|  | **N** | **Mean (SE)** | **Mean (SE)** | **Mean (SE)** | **P** |
| *Baseline* | 150 | 1.846 (0.085) | — | — | **—** |
| *Healthy* | 126 | 1.779 (0.084) | — | — | **—** |
| *Suspicious* | 15 | 1.849 (0.202) | — | — | — |
| *Cancer* | 9 | 2.781 (0.681) | — | — | — |
| *Year 1* | 94 | 1.809 (0.115) | 1.386 (0.081) | -0.423 (0.105) | **<.001** |
| *Healthy* | 76 | 1.705 (0.112) | 1.334 (0.094) | -0.371 (0.100) | **<.001** |
| *Suspicious* | 11 | 1.867 (0.252) | 1.561 (0.224) | -0.306 (0.335) | 0.382 |
| *Cancer* | 7 | 2.854 (0.832) | 1.683 (0.176) | -1.171 (0.740) | 0.165 |
| *Year 2* | 75 | 1.792 (0.114) | 2.031 (0.219) | 0.238 (0.186) | 0.205 |
| *Healthy* | 64 | 1.751 (0.123) | 1.790 (0.170) | 0.039 (0.154) | 0.802 |
| *Suspicious* | 5 | 2.077 (0.480) | 4.185 (2.261) | 2.108 (1.806) | 0.308 |
| *Cancer* | 6 | 1.994 (0.463) | 2.802 (0.761) | 0.808 (0.581) | 0.224 |
| *Year 3* | 56 | 1.821 (0.163) | 2.237 (0.156) | 0.416 (0.172) | **0.019** |
| *Healthy* | 46 | 1.685 (0.140) | 2.120 (0.161) | 0.436 (0.162) | **0.010** |
| *Suspicious* | 5 | 2.011 (0.546) | 2.387 (0.770) | 0.376 (0.432) | 0.433 |
| *Cancer* | 5 | 2.889 (1.191) | 3.162 (0.496) | 0.273 (1.276) | 0.841 |
| *Year 4* | 27 | 1.801 (0.177) | 2.520 (0.179) | 0.719 (0.217) | **0.003** |
| *Healthy* | 20 | 1.710 (0.198) | 2.323 (0.174) | 0.613 (0.221) | **0.012** |
| *Suspicious* | 3 | 2.507 (0.818) | 2.607 (0.491) | 0.100 (0.803) | 0.912 |
| *Cancer* | 4 | 1.728 (0.341) | 3.441 (0.667) | 1.714 (0.668) | 0.083 |
| *SE: standard error. P: p-value from paired t-test; Bold: the CD44 mean difference follow-up visit minus baseline is statistically significant different from zero (p<0.05).* | | | | | |

**Additional Figure 1. Mean TP in Subjects with Baseline and ≥1 Follow-Up Visit**


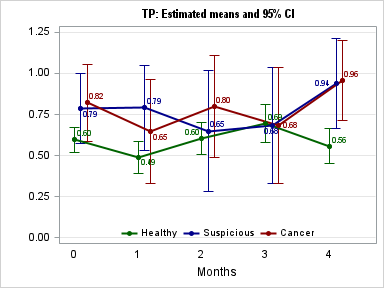


| **Additional Table 6. Unadjusted and FDR-adjusted p-values For Selected Comparisons of  Estimated TP Means** | | | | | | | |
| --- | --- | --- | --- | --- | --- | --- | --- |
| *Group 1* | | *Group 2* | | *Mean* | |  |  |
| *Visit* | *Disease Status* | *Visit* | *Disease Status* | *Difference^a^* | *SE* | *Raw-p* | *FDR-p* |
| *Baseline* | *Cancer* | *Baseline* | *Healthy* | +0.2276 | 0.1259 | 0.0737 | 0.2210 |
| *Baseline* | *Cancer* | *Baseline* | *Suspicious* | +0.0350 | 0.1613 | 0.8289 | 0.9325 |
| *Baseline* | *Suspicious* | *Baseline* | *Healthy* | +0.1927 | 0.1151 | 0.0973 | 0.2501 |
| *Year 1* | *Cancer* | *Year 1* | *Healthy* | +0.1578 | 0.1684 | 0.3511 | 0.6243 |
| *Year 1* | *Cancer* | *Year 1* | *Suspicious* | -0.1459 | 0.2084 | 0.4856 | 0.6243 |
| *Year 1* | *Suspicious* | *Year 1* | *Healthy* | +0.3037 | 0.1415 | 0.0345 | 0.1348 |
| *Year 4* | *Cancer* | *Year 4* | *Healthy* | +0.3996 | 0.1351 | 0.0066 | 0.1194 |
| *Year 4* | *Cancer* | *Year 4* | *Suspicious* | +0.0181 | 0.1876 | 0.9237 | 0.9781 |
| *Year 4* | *Suspicious* | *Year 4* | *Healthy* | +0.3814 | 0.1507 | 0.0179 | 0.1348 |
| *Year 1* | *Cancer* | *Baseline* | *Cancer* | -0.1778 | 0.1594 | 0.2667 | 0.6000 |
| *Year 2* | *Cancer* | *Year 1* | *Cancer* | +0.1539 | 0.1907 | 0.4210 | 0.6243 |
| *Year 4* | *Cancer* | *Baseline* | *Cancer* | +0.1343 | 0.1706 | 0.4335 | 0.6243 |
| *Year 1* | *Suspicious* | *Baseline* | *Suspicious* | +0.0031 | 0.1310 | 0.9812 | 0.9812 |
| *Year 2* | *Suspicious* | *Year 1* | *Suspicious* | -0.1415 | 0.1975 | 0.4751 | 0.6243 |
| *Year 4* | *Suspicious* | *Baseline* | *Suspicious* | +0.1511 | 0.1763 | 0.3949 | 0.6243 |
| *Year 1* | *Healthy* | *Baseline* | *Healthy* | -0.1079 | 0.0492 | 0.0301 | 0.1348 |
| *Year 2* | *Healthy* | *Year 1* | *Healthy* | +0.1175 | 0.0559 | 0.0374 | 0.1348 |
| *Year 4* | *Healthy* | *Baseline* | *Healthy* | -0.0376 | 0.0661 | 0.5712 | 0.6854 |
| ^a^ Group 1 minus Group 2 mean difference comparing two disease status groups at a fixed visit or two visits within a particular group of subjects; estimated mean differences and standard errors (SEs) from RANOVA model including group, visit, and group×visit interaction. | | | | | | | |

| **Additional Table 7. Unadjusted and FDR-adjusted p-values For Selected Comparisons of  Estimated TP Means** | | | | | | | |
| --- | --- | --- | --- | --- | --- | --- | --- |
| *Group 1* | | *Group 2* | | *Mean* | |  |  |
| *Visit* | *Disease Status* | *Visit* | *Disease Status* | *Difference^a^* | *SE* | *Raw-p* | *FDR-p* |
| *Baseline* | *Cancer* | *Baseline* | *Healthy* | +0.2213 | 0.1276 | 0.0860 | 0.2580 |
| *Baseline* | *Cancer* | *Baseline* | *Suspicious* | +0.0387 | 0.1624 | 0.8121 | 0.9136 |
| *Baseline* | *Suspicious* | *Baseline* | *Healthy* | +0.1826 | 0.1173 | 0.1227 | 0.3156 |
| *Year 1* | *Cancer* | *Year 1* | *Healthy* | +0.1582 | 0.1693 | 0.3524 | 0.5924 |
| *Year 1* | *Cancer* | *Year 1* | *Suspicious* | -0.1447 | 0.2091 | 0.4906 | 0.5924 |
| *Year 1* | *Suspicious* | *Year 1* | *Healthy* | +0.3030 | 0.1417 | 0.0352 | 0.1475 |
| *Year 4* | *Cancer* | *Year 4* | *Healthy* | +0.3970 | 0.1372 | 0.0076 | 0.1376 |
| *Year 4* | *Cancer* | *Year 4* | *Suspicious* | +0.0120 | 0.1895 | 0.9499 | 0.9710 |
| *Year 4* | *Suspicious* | *Year 4* | *Healthy* | +0.3850 | 0.1526 | 0.0182 | 0.1475 |
| *Year 1* | *Cancer* | *Baseline* | *Cancer* | -0.1882 | 0.1612 | 0.2451 | 0.5514 |
| *Year 2* | *Cancer* | *Year 1* | *Cancer* | +0.1534 | 0.1907 | 0.4225 | 0.5924 |
| *Year 4* | *Cancer* | *Baseline* | *Cancer* | +0.1200 | 0.1745 | 0.4936 | 0.5924 |
| *Year 1* | *Suspicious* | *Baseline* | *Suspicious* | -0.0048 | 0.1317 | 0.9710 | 0.9710 |
| *Year 2* | *Suspicious* | *Year 1* | *Suspicious* | -0.1425 | 0.1981 | 0.4733 | 0.5924 |
| *Year 4* | *Suspicious* | *Baseline* | *Suspicious* | +0.1467 | 0.1787 | 0.4150 | 0.5924 |
| *Year 1* | *Healthy* | *Baseline* | *Healthy* | -0.1251 | 0.0570 | 0.0294 | 0.1475 |
| *Year 2* | *Healthy* | *Year 1* | *Healthy* | +0.1162 | 0.0563 | 0.0410 | 0.1475 |
| *Year 4* | *Healthy* | *Baseline* | *Healthy* | -0.0557 | 0.0740 | 0.4540 | 0.5924 |
| Analysis of means from RANOVA model accounting for tobacco and age as confounders  ^a^ Group 1 minus Group 2 mean difference comparing two disease status groups at a fixed visit or two visits within a particular group of subjects; estimated mean differences and standard errors (SEs) from RANOVA model including group, visit, group×visit interaction, and tobacco and age as potential confounders. | | | | | | | |
